# Incidence and Severity of SARS-CoV-2 Infections in People With Primary Ciliary Dyskinesia

**DOI:** 10.3389/ijph.2023.1605561

**Published:** 2023-08-17

**Authors:** Eva S. L. Pedersen, Leonie D. Schreck, Myrofora Goutaki, Sara Bellu, Fiona Copeland, Jane S. Lucas, Marcel Zwahlen, Claudia E. Kuehni

**Affiliations:** ^1^ Institute of Social and Preventive Medicine, University of Bern, Bern, Switzerland; ^2^ Graduate School for Health Sciences, University of Bern, Bern, Switzerland; ^3^ Paediatric Respiratory Medicine, Children’s University Hospital of Bern, University of Bern, Bern, Switzerland; ^4^ Associazione Italiana Discinesia Ciliare Primaria Sindrome di Kartagener Onlus, Onlus, Italy; ^5^ PCD Support UK, London, United Kingdom; ^6^ Primary Ciliary Dyskinesia Centre, NIHR Biomedical Research Centre, University Hospital Southampton NHS Foundation Trust, Southampton, United Kingdom; ^7^ School of Clinical and Experimental Medicine, Faculty of Medicine, University of Southampton, Southampton, United Kingdom

**Keywords:** pandemic, chronic lung disease, rare disease, incidence, longitudinal

## Abstract

**Objectives:** There is little data on SARS-CoV-2 in people with rare chronic diseases. We studied incidence and severity of SARS-CoV-2 and its risk factors in people with primary ciliary dyskinesia (PCD) from May 2020 to May 2022.

**Methods:** We used self-reported questionnaire data from the COVID-PCD study at baseline or during weekly follow-ups. We studied factors associated with SARS-CoV-2 and symptoms using Poisson regression.

**Results:** By May 2022, 728 people participated (40% male, median age 27 years; range 0–85). 87 (12%) reported SARS-CoV-2 at baseline or during follow-up and 62 people reported an incident SARS-CoV-2 infection during 716 person-years (incidence rate 9 per 100 person years). The strongest predictors for reporting SARS-CoV-2 were exposure during periods where Delta variant was dominant (IRR 4.52, 95% CI 1.92–10.6) and Omicron variants (IRR 13.3, 95% CI 5.2–33.8). Severity was mild; 12 (14%) were asymptomatic and 75 (86%) had symptoms among whom four were hospitalized. None needed intensive care and nobody died.

**Conclusion:** The COVID-PCD study participants did not have a higher incidence of SARS-CoV-2 infections nor higher risk of severe COVID-19 disease than people from the general population.

## Introduction

Chronic respiratory disease is a risk factor for severe COVID-19, the disease caused by severe acute respiratory syndrome coronavirus 2 (SARS-CoV-2) [1–3] but evidence is still limited. Studies have shown a higher risk of intensive care unit admission and mortality in people with chronic pulmonary obstructive disease and cystic fibrosis (CF) [4–6]. Asthma, in contrast, does not seem to increase the risk for severe COVID-19 [7]. The situation remains unclear for people with rarer respiratory diseases, such as Primary Ciliary Dyskinesia (PCD). PCD is a genetic disease with a prevalence of approximately 1 in 10,000 [8, 9]. It is characterized by mutations that impair the function of motile cilia leading to chronic upper and lower airway disease and lung function impairment early in life [10–16]. To gather information about COVID-19 in people with PCD, we in May 2020 set up the COVID-PCD, an online international participatory cohort study [17]. In March 2021, we published preliminary results which showed a low incidence of 3.2 cases per 100 person-years (PY) and mainly mild disease among 24 people infected with SARS-CoV-2 [18]. Since then we have acquired more data, vaccinations against COVID-19 became available, national advice for isolation changed, and different virus variants of concern appeared, all of which influenced incidence and severity of SARS-CoV-2 infections in healthy and diseased populations [6, 19, 20].

This paper describes the incidence of SARS-CoV-2 and its risk factors in people with PCD from May 2020 to May 2022. We also describe the severity of COVID-19 disease in this population and factors associated with severity.

## Methods

### Study Design

We used data from COVID-PCD, an international participatory cohort study following people with PCD through the COVID-19 pandemic (clinicaltrials.gov: NCT04602481). COVID-PCD was initiated by people with PCD who wished to gather evidence on risk of COVID-19 in people with PCD. It is a participatory study where representatives from PCD patient support groups all over the world had a major role in developing study logistics, recruitment of study participants, and dissemination of results. COVID-PCD invites people with PCD of any age to participate. Questionnaires, based on FOLLOW-PCD [21], were adapted to three age-groups; children aged 0–13 years (completed by parents), adolescents aged 14–17, and adults aged 18 years and older. Questionnaires can be completed in five languages (English, German, Spanish, Italian, and French). Recruitment started on 30th May 2020. All participants aged 14 years and older and parents of children below 17 years gave online consent. The ethics committee of the canton of Bern, Switzerland, approved the study (Study ID: 2020-00830). This manuscript was prepared following the STROBE guidelines [22].

### Study Procedures and Questionnaires

Study procedures have been published [17]. In short, the study was advertised through PCD support groups in the United Kingdom, in North America, Switzerland, Germany, Australia, Italy, Spain, and France. Participants registered to participate through the COVID-PCD study website and then received a link via email to the baseline questionnaire which included questions on SARS-CoV-2 infections experienced prior to study entry, information about PCD diagnosis, clinical history, current symptoms, environment, and lifestyle. Participants then received weekly follow-up questionnaires with questions on incident SARS-CoV-2 infections and symptoms. In December 2021 after 80 weeks of follow-up, we reduced the frequency of follow-up questionnaires to once every 4 weeks. Participants also received an extra questionnaire focused on COVID-19 vaccinations [23]. The present paper includes all study participants who completed the baseline questionnaire.

### Definitions of Outcomes and Exposures

We defined a case as a reported positive test of SARS-CoV-2, including antigen, polymerase chain reaction (PCR), and antibody tests, reported at baseline or during follow-up. An incident case was defined as a positive test for SARS-CoV-2 reported in a follow-up questionnaire in people who had not reported an infection in the baseline-questionnaire. We defined follow-up time as days between date of study entry and date of last completed questionnaire and calculated person-years (PY) as follow-up time/365.25.

We studied the following predictors for higher risk of a reported SARS-CoV-2 infection based on findings from previous studies: age, sex, and country [24, 25], vaccination status [26, 27], and virus variant [28]. To define vaccination status and date of full vaccination, we used data from the baseline, the follow-up, and the special questionnaire on vaccinations. Date of full vaccine protection was defined as 14 days after receiving the second dose of Pfizer/BioNTech, Moderna, AstraZeneca, or Sinovac, or as 14 days after receiving first dose of Janssen, Johnson and Johnson. In participants who only reported the first dose (*n* = 38), we defined date of full vaccine protection as 44 days (1.5 months) after, assuming these people also got vaccinated a second time but did not report this date. In participants who only reported the date of the booster dose (*n* = 3), we defined date of full vaccine protection as 135 days (3.5 months) before. This interval was chosen as the booster is recommended 4 months after the second dose [29]. In our longitudinal analysis, we dichotomised vaccination status into “not fully vaccinated” until the date of full vaccination and as “fully vaccinated” after that date. We did not have information about virus variant for each infected person, and we therefore as proxy defined dominant virus variant periods based on reported time of infection. For each of the major regions: United Kingdom, Europe other than United Kingdom, North America, Australia and other regions, we divided time into periods in which one virus variant caused more than 50% of all cases based on OurWorldInData [30]. Apart from the original strain, we divided time into periods based on the three main variants of concern: Alpha, Delta, and Omicron, which were the only variants that caused more than 50% of all cases in a larger area. For simplification, we chose the closest first day in a month for the change in two periods, so that, for example, for the United Kingdom, the period dominated by the original strain went from 1 January 2020, to 31 December 2020, while the period dominated by the Alpha variant went from 1 January 2021, to 31 May 2021. The definition of each period for the major regions are described in detail in the Supplementary Table S1.

Severity of COVID-19 disease was defined by number of reported symptoms and hospitalization due to COVID-19. We did not have sufficient information to classify severity based on the world health organisation’s criteria, so as a proxy for severity grading, we created a variable representing number of reported symptoms by adding together all symptoms for each individual resulting in a count-variable from 0–16. We included the following predictors for reported symptoms based on previous literature [4, 31–33]: age, sex, bronchiectasis and self-reported FEV_1_ as proxy for severity of PCD at baseline, comorbidities, and COVID-19 vaccination. We dichotomised reported FEV_1_ as above and below 60% predicted. There is no suggested cut-off in people with PCD, however, in people with CF, an FEV_1_ below 60% has been associated with severe disease [34] and lung function impairment is comparable in people with PCD and CF [35]. Due to sample size restrictions, we could not include single comorbidities as risk factors and therefore created a composite variable defined as any of the following: hypertension, diabetes, heart disease or heart failure, cancer, Crohn’s disease, stroke, and BMI above 30. The formulation of questions to assess SARS-CoV-2, bronchiectasis, FEV_1_, and comorbidities are reported in Supplementary Table S2.

### Statistical Analyses

We described the proportion of people with PCD who reported an infection with SARS-CoV-2 as number of total SARS-CoV-2 infections reported at baseline or during follow-up divided by total number of included participants. We described incidence of reported SARS-CoV-2 as number of incident cases during follow-up per 100 PY. We computed two separate regression models, one to study predictors of reported SARS-CoV-2 infections and one to study predictors of reporting symptoms of COVID-19.

First, we studied predictors of reported SARS-CoV-2 incidence using multivariable Poisson regression with a logarithmic link function and follow-up interval in days as offset variable and report results as incidence rate ratios (IRR). As predictors, we included age stratified in groups, sex, country, vaccination status, and dominant virus variant period. Follow-up intervals for each individual correspond to the number of completed follow-up questionnaires and the interval length was days between completion of one follow-up questionnaire until completion of the next follow-up questionnaire. For each follow-up interval, reported infection with SARS-CoV-2 was defined as “No” or “Yes” corresponding to whether the person reported an infection during the follow-up interval. For our main analysis, only first infections with SARS-CoV-2 were included as the outcome because only eight people reported a second infection. In sensitivity analyses, we also included second infections as outcome and we recoded participants who only reported date of first vaccination and had missing information on date of second vaccination to “not fully vaccinated.” We included age groups in the final Poisson regression after exploring linear and non-linear effects of age as a continuous variable.

Second, to study factors associated with reported symptoms, we used multivariable Poisson regression with number of symptoms as outcome and age groups, sex, bronchiectasis, comorbidities, dominant virus variant period, and vaccination status as predictors. Due to a large proportion of missing data on FEV1, this variable was only included in a sensitivity analysis, not in the main model. We used STATA version 15 for all statistical analysis.

## Results

### Study Population

We included 728 people with PCD with a median age of 27 years (range 0–85) of whom 434 (60%) were female (Table 1). Follow-up data were available for 664 participants (90%). The median number of weeks of follow-up was 60 (range 1–100 weeks, interquartile range 26–88) and on average, participants completed 57% of the questionnaires (standard deviation 30%). One person died of reasons unrelated to SARS-CoV-2 during follow-up, and another nine people left the study (one received an alternative diagnosis than PCD and eight did not give a reason). Participants came from 48 countries with most from the United Kingdom (20%), United States (18%), and Germany (14%). At baseline, 453 (62%) reported bronchiectasis and of the 435 (60%) who reported recent lung function test results, 226 (52%) reported FEV1 below 60% predicted. The most common comorbidity was asthma reported by 25%. Most adolescents and adults were vaccinated against COVID-19; 88% of the 15–49-year-olds and 95% of those aged 50 years and older.

**TABLE 1:** Characteristics of people with primary ciliary dyskinesia included in COVID-PCD study, *N* = 728 (Switzerland, 2022).

Characteristics	Total, *N* = 728
	N (%)
Age categories	
0–14 years	228 (31)
15–49 years	381 (52)
50 years and above	119 (16)
Sex	
Male	292 (40)
Female	434 (60)
Other	2 (0)
Country	
United Kingdom	145 (20)
United States	128 (18)
Germany	103 (14)
Switzerland	48 (7)
Italy	47 (7)
France	42 (6)
Australia	32 (4)
Other European countries	123 (17)
Other non-European countries	59 (8)
Severity of PCD	
Congenital heart disease	53 (7)
Bronchiectasis	453 (62)
FEV1 percent predicted (*n* = 435)	
>60% predicted	209 (48)
<60% predicted	226 (52)
Comorbidity (at baseline)	
Hypertension^a^	38 (8)
Diabetes^a^	13 (3)
Heart disease or heart failure^a^	11 (2)
Cancer^a^	6 (1)
Crohn’s disease or Colitis ulcerosa^a^	12 (3)
Stroke ever^a^	3 (1)
BMI > 30^a^	53 (11)
Any comorbidity^a^	98 (21)
Fully vaccinated against COVID-19 (N = 569^b^)	
0–14 year-olds	79 (45)
15–49 year-olds	250 (88)
50 years or older	102 (94)

PCD, primary ciliary dyskinesia; FEV1, forced exhaled volume in 1 s, BMI, body mass index.

^a^ Only asked in adults aged 18 years or older, N = 477.

^b^ Calculated among those who completed a special questionnaire on vaccines or completed vaccination information in a follow-up questionnaire.

### Incidence of SARS-CoV-2 Infections

**FIGURE 1:**
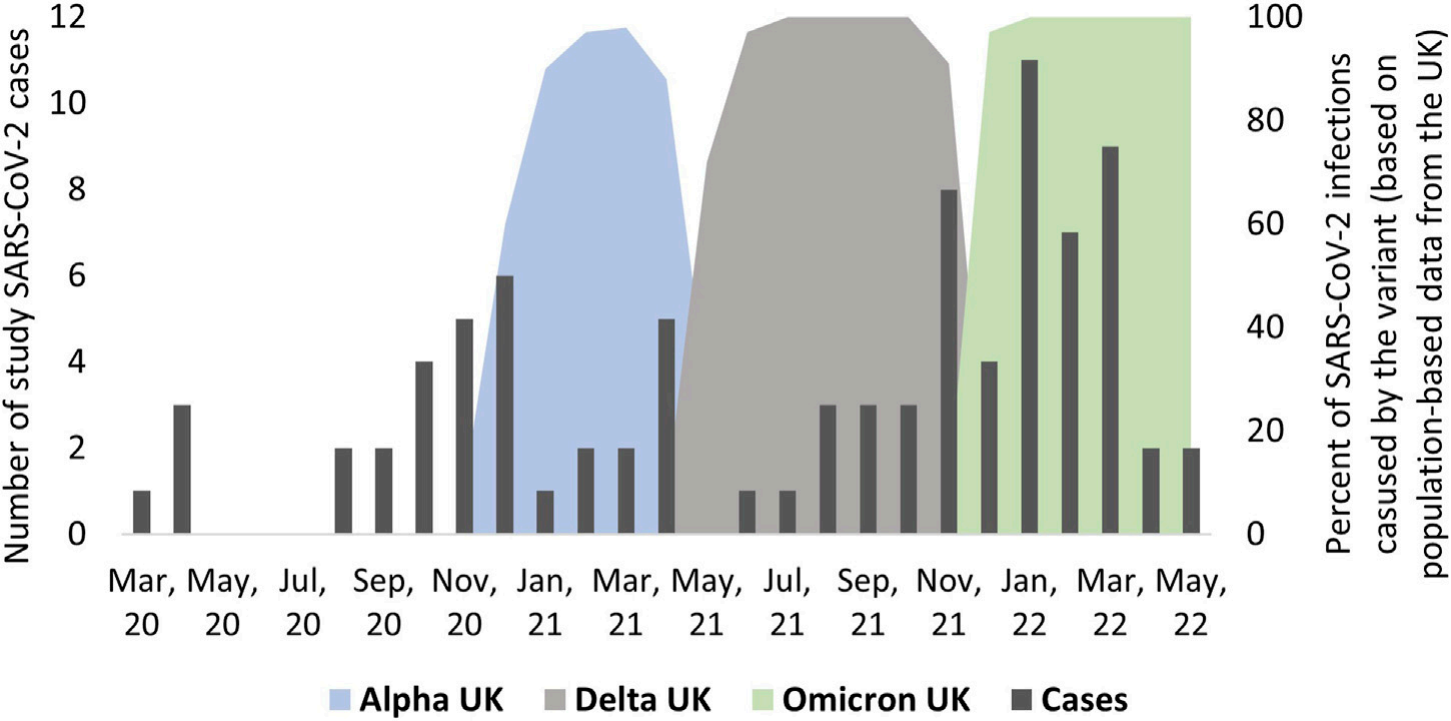
Timing of infections among people with primary ciliary dyskinesia from March 2020 to April 2022 and information about timing of dominant SARS-CoV-2 virus variants (*n* = 87), excluding re-infections (COVID-19 in people with primary ciliary dyskinesia, Switzerland, 2022).

In total, 87 participants (12%) reported a SARS-CoV-2 infection at baseline or during follow-up (Supplementary Table S3) of which 52 (60%) were confirmed by PCR test. Eight participants reported a second SARS-CoV-2 infection during follow-up. The number of infections differed between months (Figure 1). Most infections were reported during the time when Delta and Omicron were dominant. From March 2020 to May 2021, most infections were reported for adults while from May 2021 to May 2022, most infections were reported for children (data not shown). During 716 PY of follow-up, 62 participants reported a SARS-CoV-2 infection corresponding to a cumulative incidence rate of 8.7 per 100 PY [95% confidence interval (CI) 6.8–11.0] (Supplementary Table S3). In the multivariable Poisson model, we found that age above 14 years was associated with lower risk of reported infection (IRR 15–49 years 0.42, 95% CI 0.21–0.85) (Table 2). Compared to the UK, people living in other countries were less likely to report an infection (e.g., IRR Germany 0.46, 95% CI 0.21–1.03). The strongest predictors of a SARS-CoV-2 infection were infection during the period when Delta was dominant (IRR 4.52, 95% CI 1.92–10.6) and Omicron variants were dominant (IRR 13.3, 95% CI 5.2–33.8). The sensitivity analysis yielded congruent results.

**TABLE 2:** Incidence rate ratio (IRR) of SARS-CoV-2 from a Poisson regression analysis including age, country of residence, sex, vaccination status, and virus variant among people with PCD included in the COVID-PCD study, *n* = 62 (Switzerland, 2022).

	Unadjusted IRR	95% CI	Fully adjusted IRR	95% CI
Age				
0–14 years	Reference			
15–49 years	0.34	0.19–0.60	0.42	0.21–0.85
50 years or older	0.35	0.17–0.72	0.39	0.17–0.92
Country				
United Kingdom	Reference			
United States	0.44	0.17–1.10	0.52	0.21–1.32
Germany	0.73	0.35–1.54	0.46	0.21–1.03
Other European countries	0.63	0.34–1.54	0.58	0.31–1.08
Other non-European countries	0.73	0.25–2.16	0.57	0.17–1.94
Sex				
Male	Reference			
Female	0.74	0.45–1.22	0.91	0.54–1.55
Vaccination^a^				
Not fully vaccinated	Reference			
Fully vaccinated	1.16	0.68–1.96	0.62	0.29–1.34
Dominant virus variant period				
Original strain	Reference			
Alpha	2.63	1.12–6.14	2.69	1.14–6.36
Delta	3.74	1.70–8.22	4.52	1.92–10.64
Omicron	9.23	4.11–20.7	13.28	5.22–33.81

^a^ Full protection after vaccination defined as 14 days after second vaccination with vaccines requiring two shots (e.g., Pfizer, Moderna, AstraZeneca) or 14 days after vaccines requiring one shot (e.g., Janssen/Johnson and Johnson).

### Severity of SARS-CoV-2 Infections

**FIGURE 2:**
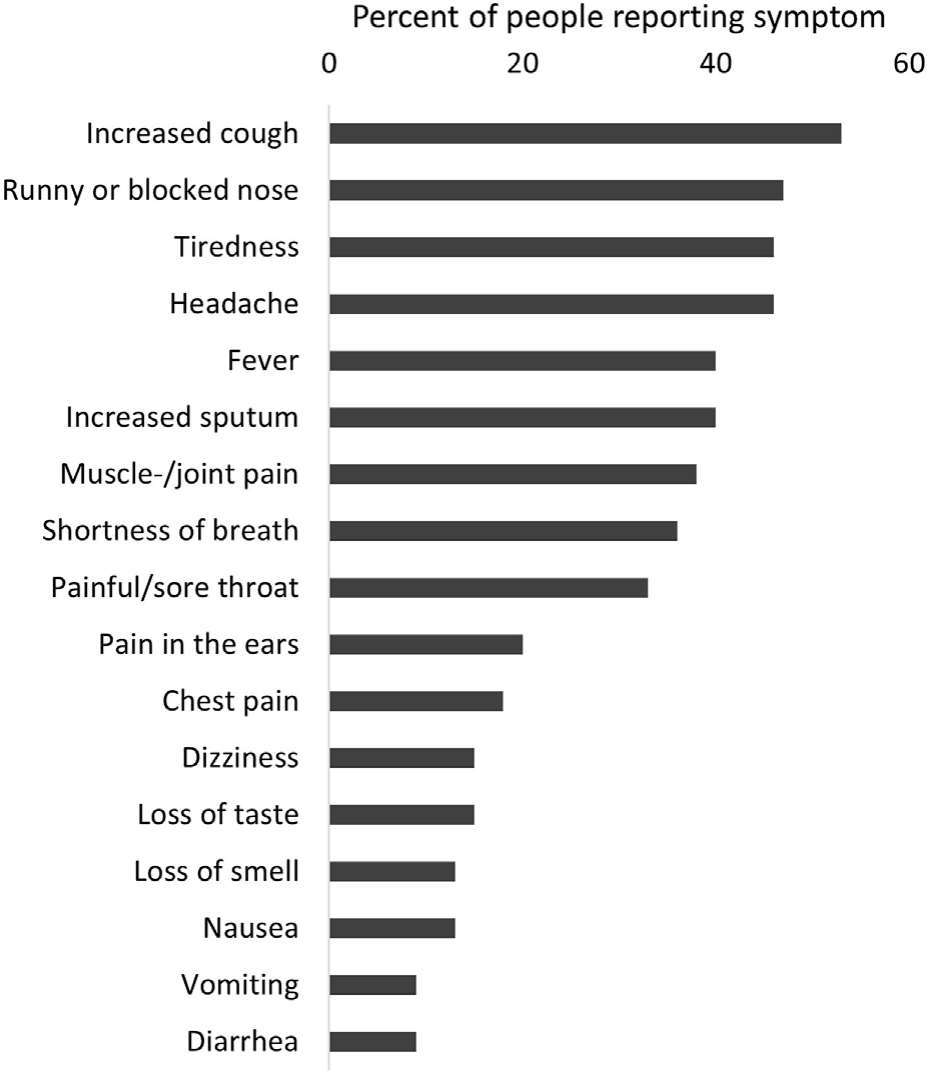
Reported symptoms among people with a SARS-CoV-2 infection (*n* = 87), excluding re-infection (COVID-19 in people with primary ciliary dyskinesia, Switzerland, 2022).

**FIGURE 3:**
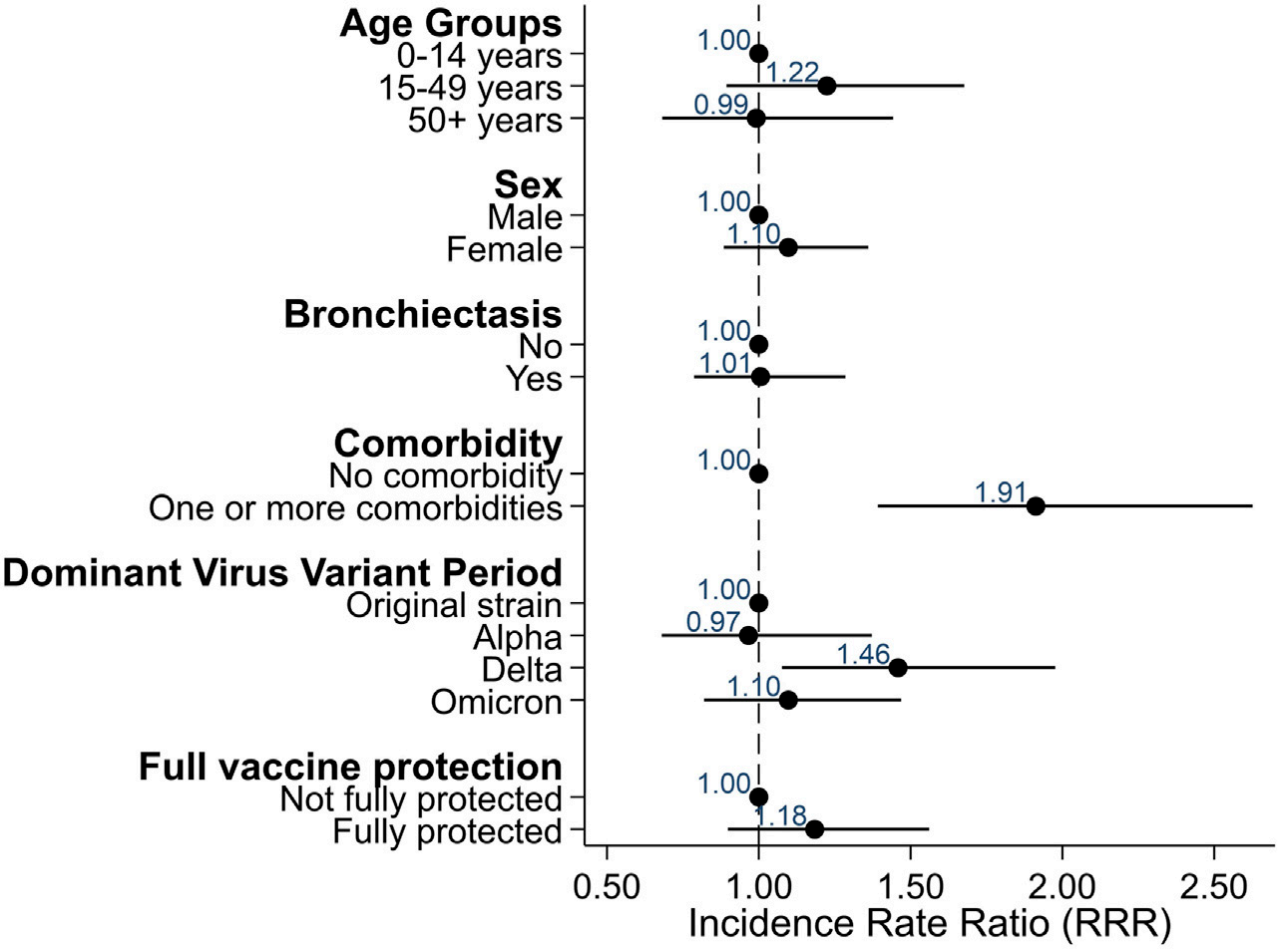
Factors associated with reporting more symptoms among COVID-PCD participants who reported a SARS-CoV-2 infection (*N* = 87), excluding re-infections (Switzerland, 2022).

Severity of disease was overall mild in the 87 participants infected with SARS-CoV-2. Twelve participants (14%) reported an asymptomatic infection, 75 (86%) reported at least one symptom Only four people reported hospitalization due to COVID-19. The longest hospital stay was 9 days, and nobody was treated in the intensive care unit or died. The most common symptoms were tiredness (60%), increased cough (61%), fever (47%), and headache (53%) (Figure 2). Using Poisson regression, we found that participants with one or more comorbidities were more likely to report more symptoms (IRR 1.91, 95% CI 1.39–2.63) as well as being infected during the period when the Delta variant was dominant (IRR 1.46, 95% CI 1.08–1.98) (Figure 3).

## Discussion

In this large participatory longitudinal study including more than 700 people with PCD, the cumulative incidence rate of reporting a SARS-CoV-2 infection was 9 per 100 PY. Children and people living in the UK were more likely to report a SARS-CoV-2 infection, and time when Delta and Omicron variants were dominant was also associated with higher likelihood of reporting an infection. Most participants reported no or few symptoms. Four participants reported hospitalization, but none were treated in the intensive care unit or died. We found that adults, participants with comorbidities, and people infected during the Delta wave were more likely to report multiple symptoms during a SARS-CoV-2 infection.

### Interpretation and Clinical Implications

The cumulative incidence of reporting a SARS-CoV-2 infection was 9 per 100 PY which was lower than what has been reported for the general population in most countries (Supplementary Table S4) [36]. In our study, we observed the highest incidence rate in France (15 per 100 PY). This corresponds also with the high cumulative incidence in the general population in France (19 per 100 PY) compared to other countries (Supplementary Table S3). Comparing incidence rate of reported SARS-CoV-2 in our study with whole-country incidence rates should however be done with caution due to differences in case definitions and methods for calculating follow-up time. The low incidence in our study population may be because people with PCD exhibited particular caution by reducing social contact, wearing masks in public, and getting vaccinated against COVID-19 [18, 23, 37]. The extraordinary effort from PCD support groups taken to inform and encourage people with PCD throughout the pandemic may also have contributed to the low incidence. The incidence rate of 9 in the present study was higher than in our first publication from the COVID-PCD study using data collected until March 2021 where we reported an incidence rate of 3.2 per 100 PY [18]. This is explained by the higher incidence rate during periods dominated by the Delta variant (IRR 4.5 in our study compared to the period with the original strain) and Omicron (IRR 13.8), and release of lockdowns and other strict public protection measures during the second year of the pandemic. The higher incidence rate during the Delta and Omicron period has also been shown for the general population [28, 38, 39]. Omicron has increased resistance to antiviral immunity, which also affects the protective effect from vaccines [40, 41]. Vaccination against COVID-19 was not associated with lower risk of reporting an infection in our study, but this is probably due to the small number of non-vaccinated participants. Most adults in our study got vaccinated (95%) during the study period, and only one unvaccinated adult reported an infection after vaccination became available. Thus, after adjusting for age and calendar time, we had too little statistical power to show an effect.

Overall, severity of COVID-19 was mild in our study population with 14% of people reporting asymptomatic infection, 5% reporting hospitalization, and no observed deaths. Several factors may explain this. First, old age is one of the strongest predictors of severe COVID-19 [42], but only eight people were older than 70 years in our population, which may be a reason why we observed no intensive care admissions or deaths. Secondly, 40% of the SARS-CoV-2 infections were reported during the Omicron-dominant period, which has been associated with less severe disease [32, 39]. Results from our study suggest that compared to country-specific cumulative incidence rates, people with PCD in our study may not be at higher risk of severe COVID-19 disease than people from the general population. However, although large for a rare disease, our study population is small compared to other studies, and it is difficult to assess generalizability of results from COVID-PCD participants to all people living with PCD. It is difficult to compare our results with studies in other chronic respiratory diseases because no other similar longitudinal observational study exists.

We found that only comorbidity and dominant variant period predicted COVID-19 symptoms which is in line with risk factors identified in other studies [31–33]. We found no association with demographic or clinical characteristics such as PCD severity. This is probably because the severity of the reported SARS-CoV-2 cases was overall mild in our study. Even the four people reporting hospitalisation had mild symptoms. Other studies mainly described risk factors for intensive care treatment and mortality, outcomes which we did not observe.

### Strengths and Limitations

A major strength of the study was the large study population for such a rare disease. The COVID-PCD is the largest study worldwide to collect data directly from people with PCD, and it represents all age groups and people with PCD from most places in the world. Another strength was the long follow-up starting in May 2020 and ending in May 2022. The weekly questionnaires minimized the risk that study participants would forget to report an infection with SARS-CoV-2. One reason for the high commitment of study participants to complete study questionnaires was that PCD support groups regularly encouraged members to participate in the study and keep completing questionnaires. A limitation of the study is that we only had self-reported data on test results for SARS-CoV-2. COVID-PCD is an observational study, and participants were not regularly tested for COVID-19 which may have led to an underestimation of the proportion of cases, due to unreported asymptomatic or mild cases. This would however not have affected the overall observed severity, as it is unlikely that a person with moderate or severe symptoms would not have been tested.

### Conclusion

At the start of the pandemic, it was unclear if people with PCD were at high risk of severe COVID-19 disease. In this population of people with PCD, the incidence of reported SARS-CoV-2 was lower than in the general population and severity mild. This may well be due to the particular caution that people with PCD took to avoid infection and getting vaccinated against COVID-19.

## Data Availability

The COVID-PCD data is available on reasonable request by contacting CK by email: claudia.kuehni@unibe.ch.

## Group Members of COVID-PCD Patient Advisory Group

Sara Bellu, Associazione Italiana Discinesia Ciliare Primaria Sindrome di Kartagener Onlus Italy; Isabelle Cizeau, Association ADCP, France; Fiona Copeland, PCD support UK; Katie Dexter, PCD support UK; Lucy Dixon, PCD support UK; Trini López Fernández, Asociación Española de Pacientes con Discinesia Ciliar Primaria, Spain; Susanne Grieder, Selbsthilfegruppe Primäre Ciliäre Dyskinesie, Switzerland; Catherine Kruljac, PCD Australia Primary Ciliary Dyskinesia, Australia; Michelle Manion, PCD Foundation, United States; Bernhard Rindlisbacher, Selbsthilfegruppe Primäre Ciliäre Dyskinesie, Switzerland; Hansruedi Silberschmidt, Verein Kartagener Syndrom und Primäre Ciliäre Dyskinesie, Germany.
