# Supplementary material for: Incidence and Severity of SARS-CoV-2 Infections in People With Primary Ciliary Dyskinesia
Source: Int J Public Health. 2023 Aug 17;68:1605561. doi: 10.3389/ijph.2023.1605561 (PMC10470037; doi:10.3389/ijph.2023.1605561)
Supplement: Supplementary file 1 [file DataSheet1.docx]

**Supplementary table 1: Definition of periods dominated by SARS-CoV-2 virus variants**

| **Region** | **Original strain** | **Alpha** | **Delta** | **Omicron** |
| --- | --- | --- | --- | --- |
| **UK** | Jan 2020 – December 31, 2020 | January 1, 2021 – May 31, 2021 | June 1, 2021 – December 31, 2021 | January 1, 2022 onwards |
| **Europe other than UK** | Jan 2020 – January 31, 2021 | February 1, 2021 – June 30, 2021 | July 1, 2021 – December 31, 2021 | January 1, 2022 onwards |
| **North America** | Jan 2020 – January 31, 2021 | February 1, 2021 – June 30, 2021 | July 1, 2021 – December 31, 2021 | January 1, 2022 onwards |
| **Australia and other regions** | Jan 2020 – January 31, 2021 | February 1, 2021 – June 30, 2021 | July 1, 2021 – December 31, 2021 | January 1, 2022 onwards |

**Supplementary table 2: Formulation of questions on SARS-CoV-2 infections, clinical characteristics, and co-morbidities in adults participating in COVID-PCD.**

| **Baseline** | **Question** | **Answer categories** |
| --- | --- | --- |
| COVID-19 ever | Have you ever had COVID-19? | No; yes once; yes twice; yes three times; I don’t know |
| Date of positive test | What date did you test positive the first time? (If you cannot remember the exact date, please select the date you think is correct) (asked for up to three time depending on the answer of question above) | date_dmy |
| Tested for COVID-19 | Have you been tested for COVID-19? | No; yes |
| Type of COVID-19 test | Which type of COVID-19 test was performed? | Viral test to identify a current COVID-19 infection usually done using a swab in the throat or nose; Antibody test to identify a past COVID-19 infection done by a blood test; Rapid antigen test to identify a current COVID-19 infection done using a swab in the throat or nose that gives a result within an hour; I don’ t know |
| COVID-19 test result | Was the test positive? | No; Yes; I don’t know |
| COVID-19 test date | What date were you tested for COVID-19? (If you cannot remember the exact date, please select the date you think is correct) | date_dmy |
| Severity of symptoms | How seriously ill did you get during your COVID-19 infection? | No symptoms (asymptomatic); Mild symptoms (e.g. mild fever and/or cough); Severe symptoms (e.g. high fever, cough, headache, etc.) |
| Symptom | For this question, we would like you to think about symptoms that you experienced during your infection: Did you notice a worsening or new occurence of the symptoms listed below during your COVID-19 infection? (Tick all that apply) | Fever/temperature; Chills; Muscle- /joint pain; Headache; Confusion; Increased cough; Increased sputum; Shortness of breath; Chest tightness; Chest pain; Sneezing; Runny/blocked nose; Loss of smell; Pressure or pain in the ears; Sore, scratchy, or painful throat; Difficulty swallowing; Loss of taste; Watery, red eyes; Tiredness or exhaustion; Loss of appetite; Dizziness; Dizziness; Nausea; Vomiting; Diarrhoea; Rash; Other symptoms |
| Hospitalization | Have you been in hospital (one night or more) because of COVID-19? | No; Yes |
| Hospitalization reason | Were you hospitalised because of COVID-19 or because of another reason? | I was hospitalised because of COVID-19; I was hospitalised for another reason (for example another infection or surgery), and the COVID-19 infection was detected with routine testing |
| Date of hospitalisation |  |  |
| COVID-19 vaccination | Have you been vaccinated against COVID-19? | Yes, I received one/two/three/four doses |
| Type of COVID-19 vaccine | Which vaccine did you get? | Pfizer-BioNTech (BNT162b2); Moderna (mRNA-1273); AstraZeneca, also called Oxford vaccine (AZD1222); Janssen/Johnson & Johnson (Ad26.COV2.S.); Sputnik V; CoronavAC (Sinovac); BBIBP-CorV (Sinopharm); EpiVacCorona; Convidicea (Ad5nCov) (CanSino Biologics); Covaxin (Bharat Biotech); Other; I don't know |
| Date of vaccination | What date did you get the first/second/third/fourth dose of vaccine? (If you cannot remember the exact date, please select the date you think is correct) | date_dmy |
| Bronchietasis | Have you been diagnosed with bronchiectasis? (Bronchiectasis is a widening of the bronchi or airways caused by PCD, which can be seen in tests such as chest X-ray or CT (computer tomography) scans or MRI (magnetic resonance) examinations) | No; Yes; I don’t know |
| Lung function performed | Have you had your lung function measured in the past year? (By this we mean that you have blown through a tube and a computer has recorded your blow. This can be done in open space, or in a closed cabin) | No; Yes; I don’t know |
| Result of FEV1 | What was the last FEV1? (This is the amount of air that can be blown out in 1 second; and is used to monitor lung function over time) | better than 90% predicted; 80-90% predicted; 70-80% predicted; 60-70% predicted; 50-60% predicted; below 40% predicted, I don’ t know |
| Asthma | Have you been diagnosed with asthma by a doctor and currently receive treatment? | No; Yes |
| Hypertension | Are you currently being treated for hypertension/high blood pressure? | No; Yes |
| Diabetes | Are you currently being treated for diabetes? | No; Yes |
| Heart disease | Are you currently being treated for heart disease or heart failure or have you had a myocardial infarction (heart attack) or coronary artery disease? | No; Yes |
| Cancer | Are you currently being treated for cancer? | No; Yes |
| IBD | Do you have inflammatory bowel disease (e.g. Crohn's disease or Colitis ulcerosa)? | No; Yes |
| Stroke | Have you ever had a stroke? | No; Yes |

**Supplementary table 3**: **Proportion of infected participants and incidence rate (excluding re-infections) among people with primary ciliary dyskinesia in the COVID-PCD study (N=728) by age, sex, and country.**

|  | **N** | **Infected with SARS-CoV-2, n (%)** | **Incident infections (reported during follow-up)** | **Follow-up time (in person-years)** | **Incidence rate (infections per 100 person years, 95% CI)** |
| --- | --- | --- | --- | --- | --- |
|  |  |  |  |  |  |
| **Total population** | 728 | 87 (12) | 62 | 716 | 9 (7-11) |
| **Age groups** |  |  |  |  |  |
| 0-14 years | 228 | 42 (18) | 35 | 218 | 16 (12-22) |
| 15-49 years | 381 | 32 (8) | 18 | 341 | 5 (3-8) |
| 50 years or more | 119 | 13 (11) | 9 | 157 | 6 (3-10) |
|  |  |  |  |  |  |
| **Sex** |  |  |  |  |  |
| Male | 292 | 39 (13) | 28 | 271 | 10 (7-15) |
| Female | 434 | 48 (11) | 34 | 443 | 8 (6-11) |
|  |  |  |  |  |  |
| **Country** |  |  |  |  |  |
| United Kingdom | 145 | 19 (13) | 19 | 159 | 12 (8-18) |
| USA | 128 | 13 (10) | 6 | 112 | 5 (3-12) |
| Germany | 103 | 18 (18) | 11 | 122 | 9 (5-16) |
| Switzerland | 48 | 2 (4) | 2 | 54 | 4 (1-14) |
| Italy | 47 | 3 (6) | 2 | 38 | 5 (1-20) |
| France | 42 | 6 (14) | 4 | 27 | 15 (6-37) |
| Australia | 32 | 3 (9) | 3 | 32 | 9 (3-28) |
| Other European countries | 123 | 16 (13) | 11 | 128 | 9 (5-15) |
| Other non-European countries | 59 | 6 (10) | 4 | 44 | 9 (3-23) |

**Supplementary table 4:** Proportion of infected people and cumulative incidence rate in the general population by 10.05.2022 for countries represented (by at least 10 persons) in COVID-PCD

| **Country/area** | **Follow-up start date*** | **Follow-up years (in millions)** | **Population size** | **Total nr. cases** | **Cumulative IR per 100 person years** |
| --- | --- | --- | --- | --- | --- |
| **COVID-PCD** | 30.05.2022 | 0.007 | 728 | 62 | 9 |
| **Australia** | 26.01.2020 | 58.9 | 25788217 | 6392018 | 10.9 |
| **Canada** | 23.01.2020 | 87.2 | 38067913 | 3814635 | 4.4 |
| **Denmark** | 02.02.2020 | 13.1 | 5813302 | 3124899 | 23.9 |
| **France** | 24.01.2020 | 154.3 | 67422000 | 29075412 | 18.8 |
| **Germany** | 27.01.2020 | 191.3 | 83900471 | 25503878 | 13.3 |
| **Ireland** | 29.02.2020 | 10.9 | 4982904 | 1527328 | 14.0 |
| **Italy** | 31.01.2020 | 137.0 | 60367471 | 16872618 | 12.3 |
| **Netherlands** | 27.02.2020 | 37.7 | 17173094 | 8073152 | 21.4 |
| **Norway** | 12.02.2020 | 12.2 | 5465629 | 1428699 | 11.7 |
| **Spain** | 01.02.2020 | 106.1 | 46745211 | 12058888 | 11.4 |
| **Switzerland** | 25.02.2020 | 19.2 | 8715494 | 3649155 | 19.0 |
| **USA** | 22.01.2020 | 763.8 | 332915074 | 82087703 | 10.7 |
| **United Kingdom** | 31.01.2020 | 154.8 | 68207114 | 22225565 | 14.4 |
| **Europe** | 23.01.2020 | 1716.3 | 748962983 | 195099900 | 11.4 |
| **North America** | 22.01.2020 | 1368.7 | 596581283 | 97151945 | 7.1 |

Data source: our world in data (<https://ourworldindata.org/covid-cases>, data downloaded on 11.06.2022). *Follow-up end date was set to 10.05.2022, same as in our study. Start date was defined as date of first case observed. Follow-up years was calculated as time between start date and end date times population size assuming that all persons were followed up the entire period of time.
